# Intranasal BCG vaccination induces systemic and pulmonary mucosal immune responses against tuberculosis in a goat model

**DOI:** 10.3389/fimmu.2025.1740197

**Published:** 2026-01-02

**Authors:** Patricia Cuenca-Lara, Miriam Blay-Benach, Zoraida Cervera, Julia Moraleda, Iker A. Sevilla, Joseba M. Garrido, Mahavir Singh, Sergio López-Soria, Enric Vidal, Mariano Domingo, Bernat Pérez de Val

**Affiliations:** 1 Unitat Mixta d’Investigació Institut de Recerca i Tecnologia Agroalimentàries (IRTA)-UAB en Sanitat Animal, CReSA, Campus de la Universitat Autònoma de Barcelona (UAB), Bellaterra, Catalonia, Spain; 2 Institut de Recerca i Tecnologia Agroalimentàries (IRTA), Animal Health, Centre de Recerca en Sanitat Animal (CReSA), Campus de la Universitat Autònoma de Barcelona (UAB), Bellaterra, Catalonia, Spain; 3 Animal Health Department, NEIKER-Instituto Vasco de Investigación y Desarrollo Agrario, Basque Research and Technology Alliance (BRTA), Derio, Bizkaia, Spain; 4 Lionex Diagnostics and Therapeutics GmbH, Braunschweig, Germany; 5 Departament de Sanitat i Anatomia Animals, Facultat de Veterinària, Campus de la Universitat Autònoma de Barcelona (UAB), Bellaterra, Barcelona, Spain

**Keywords:** alveolar macrophages, animal model, BCG, goat, heat-inactivated *Mycobacterium bovis*, mucosal immunity, mucosal vaccines, tuberculosis

## Abstract

Early immune containment of mycobacteria at the infection site is key to tuberculosis (TB) vaccine development. Intranasal delivery strategies offer a promising alternative to parenteral BCG vaccination, particularly for pulmonary TB, the predominant clinical form in humans and livestock. This study evaluated the immunogenicity of intranasal BCG and heat-inactivated *M. bovis* (HIMB) with or without adjuvant, as well as prime-boost strategies combining parenteral BCG or HIMB followed by intranasal HIMB in young goats. Intranasal BCG elicited systemic antigen-specific IFNγ production, with enhanced expansion of CD4^+^IFNγ^+^ and CD8^+^IFNγ^+^ T-cells, comparable to prime-boost regimens. Intranasal BCG and prime-boosted groups also induced higher local proinflammatory responses at the lung mucosa, including proinflammatory cytokine production, expansion of antigen-specific T-cells, and polarization of alveolar macrophages toward activated proinflammatory phenotype. The results underscore the potential of respiratory mucosal BCG delivery to enhance early immune responses against TB infection and support further investigation into its protective efficacy.

## 1 Introduction

Tuberculosis (TB) is a chronic infectious disease caused by members of the *Mycobacterium tuberculosis* complex (MTBC). Despite being a preventable and curable disease, TB remains as the world’s leading cause of death from a single infectious agent in humans (1). In animals, a wide range of mammals act as reservoirs of TB, including domestic species such as cattle and goats, as well as wildlife (2). While *M. tuberculosis* is the main causative agent of human TB, animal TB is primarily attributed to *M. bovis*, *M. caprae*, and *M. microti*, which can also infect humans and vice versa, being then a zoonosis (3).

The *Bacillus* Calmette-Guérin (BCG), a live-attenuated strain of *M. bovis*, remains the only licensed vaccine against TB for humans (4, 5), however, it has variable efficacy and does not fully prevent TB infection in adults (6). Similarly, BCG administered parentally and orally has shown a variable degree of protection against MTBC in cattle, goats, and wildlife (7–12). An alternative approach of animal vaccination using a heat-inactivated *M. bovis* (HIMB) vaccine administered parentally and orally has also shown divergent levels of protection against MTBC challenge in various species (13–17). Given these limitations, there is a clear need to explore novel vaccine candidates and strategies that can elicit stronger and more consistent protection across hosts.

To date, the understanding of protective immunity against TB in both animals and humans remains incomplete and correlates of protection are poorly defined (18), as efficient immunity may comprehend a complex interplay between adaptive and innate responses. Antigen-specific CD4^+^ T-cell subsets producing IFNγ and TNFα have been identified as key components of protective immunity (19, 20). However, BCG and HIMB vaccines, when administered by subcutaneous or intradermal routes, induce systemic T-cell-specific responses that are insufficient to confer full protection against MTBC challenge (16, 21–23), suggesting that additional immune mechanisms are required to effectively prevent infection.

Given that MTBC is primarily transmitted via aerosols and enters the host through the airway mucosa, it is hypothesized that vaccine efficacy could be improved by changing administration route and harnessing mucosal immunity mechanisms (24–27). Mucosal vaccination with BCG via the respiratory tract has been reported to elicit robust antigen-specific memory CD4^+^ and CD8^+^ T-cell responses in the airways, resulting in enhanced protection (28–31). Moreover, mucosal vaccination has also been shown to activate innate immune mechanisms within the lung mucosa, particularly alveolar macrophages (AMs), associated with reduced MTBC infection (32, 33).

**Figure 1:**
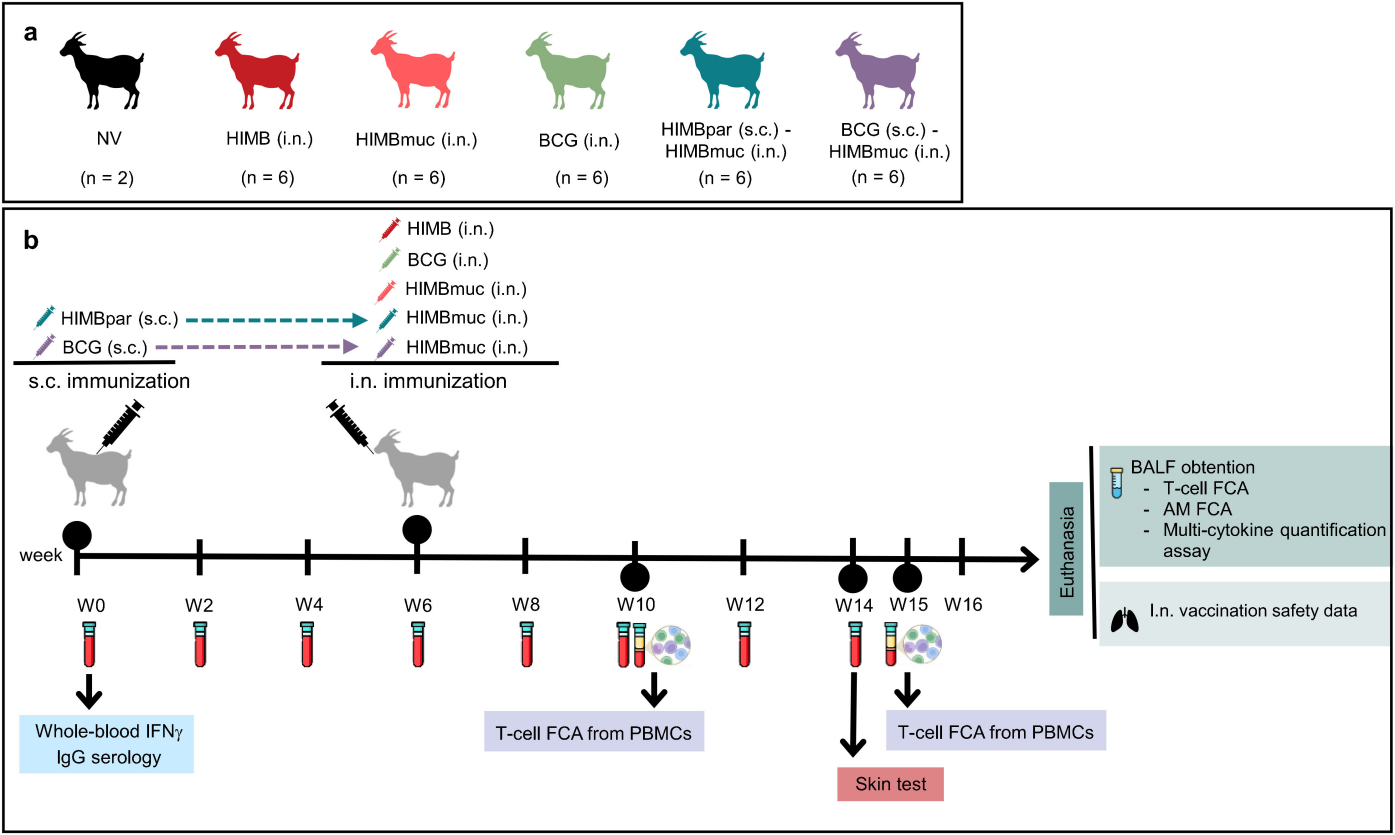
Experimental design **(a)** Vaccines and administration methods; NV, non-vaccinated animals; intranasal (i.n.) *Bacillus* Calmette-Guérin (BCG) at 2–3 x 10^7^ CFU/ml; i.n. heat-inactivated *Mycobacterium bovis* (HIMB) at 2 x 10^8^ CFU/ml and subcutaneous (s.c.) HIMB at 8 x 10^7^ CFU/ml, and s.c. BCG at 10^5^ CFU/ml; HIMBmuc (i.n.) was administered with 20% Montanide™ GEL 02 PR adjuvant (Seppic, Paris, France); HIMBpar (s.c.) was administered as 36% of HIMB suspension and 64% Montanide™ ISA 61 VG (Seppic) adjuvant **(b)** Experimental procedures. The subcutaneous prime vaccination was performed at week 0 with a HIMBpar (HIMBpar (s.c.) - HIMBmuc (i.n.) group) and BCG vaccine (BCG (s.c.) - HIMBmuc (i.n.) group); at week 6 was performed the intranasal vaccination with BCG (BCG i.n.), HIMBmuc (HIMBmuc i.n. group), HIMB without adjuvant (HIMB i.n. group), and intranasal boost vaccination with HIMBmuc (HIMBpar (s.c.) - HIMBmuc (i.n.) group; and BCG (s.c.) - HIMBmuc (i.n.) group). FCA, Flow cytometry assay; BALF, bronchoalveolar lavage fluid; AM, alveolar macrophage.

In this study, we aimed to assess the immunogenicity of different single-dose and prime-boost vaccination strategies using intranasal BCG or HIMB (with or without adjuvant) vaccines in a goat model, following the experimental design outlined in **Figure 1**. We assessed MTBC-specific immune responses both in peripheral blood and at the pulmonary mucosal level.

## 2 Materials and methods

### 2.1 Experimental animals and study design

Thirty-two goat kids of approximately 4 months of age, of the Murciano-Granadina breed were used in this study. A graphical outline of the vaccination groups and the experiments performed is shown in **Figures 1a, b**. Two animals were used as unvaccinated controls (NV). The remaining thirty animals were randomly divided into five groups of six animals each (3 males and 3 females): (1) Intranasal *M. bovis* Bacillus Calmette-Guérin (BCG i.n.); (2) I.n. heat-inactivated *M. bovis* (HIMB i.n.); (3) I.n. HIMB with mucosal adjuvant (HIMBmuc i.n.); (4) Priming with subcutaneous HIMB with parenteral adjuvant and boosting with i.n. HIMBmuc after six weeks (HIMBpar s.c. – HIMBmuc i.n.; (5) Priming with s.c. BCG and boosting with i.n. HIMBmuc after six weeks (BCG s.c. – HIMBmuc i.n.). Priming immunizations were carried out at week 0 whereas boosting and single-dose vaccinations were carried out at week 6.

Prior to study initiation, all animals were confirmed negative for TB by the and IFNγ release assay (IGRA, ID Screen^®^ Ruminant IFNg, ID, Grabels, France). Tuberculin skin tests were performed in all goats at week 14 of the experiment following the standard protocols to verify diagnostic outcomes (34).

The study was conducted at the *Servei de Granges i Camps Experimentals* (SGCE) of the Autonomous University of Barcelona (Registration No. B9900042). Animals were housed in two separate pens based on sex, with 16 males and 16 females. Clinical status was monitored daily, and body weight was recorded biweekly throughout the experimental period. Rectal temperatures were measured immediately before and then at 6, 24, and 48 hours following intranasal vaccination. Blood samples (10–20 ml) were collected from the jugular vein every two weeks from the start until the conclusion of the study. At week 16, animals were euthanized humanely via intravenous injection of pentobarbital (200 mg/kg).

### 2.2 Vaccines and administration methods

#### 2.2.1 BCG

The s.c. and i.n BCG used strain was the *M. bovis* BCG Danish 1331 (ATCC35733), and the preparation was performed as previously described (35). BCG was diluted in phosphate-buffered saline (PBS) to reach suspensions of ~10^5^ colony forming units (CFU)/ml for the s.c. vaccination, and 2–3 x 10^7^ CFU/ml for the i.n. administration. One ml of the first suspension was injected subcutaneously in the right scapular area and 1 ml of the second suspension was administered intranasally, with 0.5 ml delivered each nostril using a syringe-adapted nebulizing cannula (MADgic^®^, Teleflex, Morrisville, NC, USA).

#### 2.2.2 HIMB vaccines

HIMB was produced at NEIKER (Derio, Bizkaia, Spain) with approximately 2 x 10^8^ CFU/ml of a heat-treated *M. bovis* field strain (SSB0339) for the intranasal administration and 8 x 10^7^ CFU/ml for the subcutaneous administration, as previously described (15). HIMB (i.n.) was administered without adjuvant. HIMBmuc (i.n.) was prepared as an emulsion containing 20% of the Montanide™ GEL 02 PR adjuvant (Seppic, Paris, France), while HIMBpar (s.c.) was prepared an emulsion of 36% HIMB suspension and 64% Montanide™ ISA 61 VG adjuvant (Seppic). Intranasal and subcutaneous HIMB vaccines were administrated as described above for BCG i.n. and s.c. equivalents.

### 2.3 Antigens and peptides

*M. bovis* and *M. avium* purified protein derivative (PPDB and PPDA, respectively) (25,000 IU/ml) were obtained from CZ Vaccines (O Porriño, Galicia, Spain). The MTBC-specific ESAT-6, CFP10 and EspC (Rv3615c) recombinant proteins were obtained from Lionex (Braunschweig, Germany) and used as a 1:1:1 mixture at 500 µg/ml each to formulate a defined antigen cocktail (DAC). The MPB83 recombinant protein was also purchased to Lionex.

### 2.4 *In vitro* IFNγ release assay

Whole-blood samples were obtained from the jugular vein in heparinized blood tubes from the animals every two weeks. One ml of whole blood was stimulated in in 2.2 ml 96-well cell culture plates (Eppendorf, Hamburg, Germany) with PBS, PPDB and PPDA at final concentrations of 20 ug/ml each and another 225 µl of whole blood was stimulated in 300 µl 96-well cell culture plates (Thermo Fisher Scientific, Waltham, MA, USA) with DAC at a final concentration of 30 ug/ml (10 ug/ml of each antigen -ESAT-6, CFP-10 and EspC-). Blood samples were incubated overnight at 37°C with 5% CO_2_ and plasma supernatants were collected after centrifugation at 1260 g for 10 min and stored at -20°C until further analysis. An IFNγ enzyme-linked immunosorbent assays (ELISA) was performed on the thawed plasma samples using the ID Screen^®^ Ruminant IFNg kit (ID), following manufacturer’s instructions. ELISA results were obtained as optical density (OD) determined at 450 nm using a spectrophotometer (Biotek Power Wave XS^®^, Agilent, Santa Clara, CA, USA). IFNγ levels were calculated as mean PPDB OD – PBS OD (ΔOD) or DAC OD – PBS OD (ΔOD).

### 2.5 Antibody detection assay

Plasma samples were analyzed in duplicate to monitor antibody response against MTBC every two weeks. An indirect ELISA was used to measure total IgG antibodies targeting MPB83 antigen, following the protocol previously described (36). MPB83-specific IgG levels were expressed as the difference in optical density (ΔOD) at 450 nm between coated and uncoated wells.

### 2.6 Isolation of peripheral blood mononuclear cells

At week 10 and week 15, PBMCs were isolated from blood samples using the BD vacutainer CPT™ (BD, Franklin Lakes, NJ, USA), following manufacturer’s instructions with a modification: the layer above gel barrier was collected, diluted with PBS up to 50 ml, and centrifuged at 450 g for 10 minutes. The supernatant was discarded, and erythrocytes were lysed by adding 9 ml of ultrapure water and 3 ml of 3.5% NaCl for 20–30 seconds. After a second centrifugation, the supernatant was discarded, and the cells were resuspended in RPMI medium.

### 2.7 Obtention and preparation of bronchoalveolar lavage fluid

Immediately after euthanasia, the trachea was ligated cranially to avoid entry of ruminal contents and subsequently the lungs were removed. Lavage was performed by pouring 500 ml of sterile PBS with 0,1% gentamicin into the lungs via a funnel inserted into the trachea. Lungs were lightly massaged before decanting the BALF into a container. BALF was processed by centrifugation at 380 g for 15 min at 4°C with two washes and finally the pellet was resuspended in 50 ml of PBS. Cells were counted and cryopreserved in 700 µl of Cryostore and stored in liquid nitrogen for further use.

### 2.8 Peripheral blood and BALF T-cell flow cytometry assay

Freshly isolated PBMCs (10^6^ cells/well) and thawed (37°C) cells isolated from BALF (10^6^ cells/well) were incubated in 96-well plates with either medium alone or stimulated with PPDB (10 μg/ml) in RPMI cell culture medium supplemented with 10% fetal calf serum, 1% glutamine plus penicillin+streptomycin, and 0,5% nystatin (cRPMI). Cells were incubated at 37°C with 5% CO_2_, 95% humidity for 6 h. Then, Brefeldin A (BFA, Sigma-Aldrich, St. Louis, MO, USA) was added at a final concentration of 10 μg/ml, and cells were further incubated for 16 h. Cells were stained with the antibody clone, source and fluorochrome listed in **Supplementary Table S1**. Stained cells were permeabilized using the Leucoperm™ reagent kit (Bio-Rad, Hercules, CA, USA) and intracellularly stained with conjugated monoclonal antibody anti-IFNγ. Cells were finally washed and fixed with 1% paraformaldehyde and analyzed within 24 h by flow cytometry in MACSQuantify™ instrument (Milteny Biotec, Bergisch Gladbach, Germany). The gating strategy is shown in **Supplementary Figure S1** for peripheral blood T-cell cytometry and in **Supplementary Figure S2** for BALF T-cell cytometry. To analyze antigen-specific T-cell responses expression levels in unstimulated samples were subtracted from those measured in PPDB-stimulated samples.

Parallelly to the peripheral blood flow cytometry assay, isolated PBMCs were stimulated as explained above (PPDB 10 μg/ml or cRPMI), but after 16 h stimulation period, cell supernatants were collected and ELISA was performed using the ID Screen^®^ Ruminant IFNg kit, for the detection of IFNγ produced by the isolated PBMCs.

### 2.9 AMs flow cytometry assay

Isolated BALF cells were thawed at 37°C and incubated in 96-well plates (10^6^ cells/well) in cRPMI. Cells were incubated at 37°C with 5% CO_2_, 95% humidity for 6h. Then, BFA was added at a final concentration of 10 μg/ml, and cells were further incubated overnight. Next day PBS was added, and cells were incubated at room temperature (RT) for 20 min. Cells were detached for the culture plate carefully pipetting and transferred to 96-well round bottom plates. AMs were stained with the antibody clone, source and fluorochrome listed in **Supplementary Table S1**. Stained AMs were permeabilized with Leucoperm and intracellularly stained with conjugated monoclonal antibody anti-iNOS (inducible nitric oxide synthase). Cells were finally washed and fixed with 1% paraformaldehyde and analyzed within 24 h. Stained cells were analyzed by flow cytometry in MACSQuantify™ instrument (Milteny Biotec). The gating strategy is shown in **Supplementary Figure S3**.

### 2.10 BALF multi-cytokine quantification assay

Isolated cells were thawed at 37°C and stimulated in 24-well culture plates (10^6^ cells/ml) with the HIMB vaccine (MOI 10, 10^7^cfu/ml) or cRPMI as control, and incubated overnight at 37°C with 5% CO_2_, 95% humidity. Cell supernatants were collected and frozen at -20°C for further analysis. Cytokines were quantified from BALF cells supernatants after stimulation using a bovine customized multiplex assay kit (MILLIPLEX^®^ Millipore, Merck Life Science S.L.U., Madrid, Spain) including a five-plex panel for IL-1β, TNFα, IL-6, IL-10 and IL-17A. 25 µl of each plasma sample and cytokine standards were analyzed following the manufacturer’s instructions using xMAP^®^ technology (ThermoFisher Scientific). ELISA plates were read on MAGPIX instrumental platform with xPONENT acquisition software (ThermoFisher Scientific).

### 2.11 BALF IFNγ quantification assay

IFNγ enzyme-linked immunosorbent assays (ELISA) was performed on the thawed BALF samples using the ID Screen^®^ Ruminant IFNg kit (ID), following manufacturer’s instructions. ELISA results were obtained as optical density (OD) determined at 450 nm using a spectrophotometer (Biotek Power Wave XS^®^, Agilent, Santa Clara, CA, USA). The positive control provided with the kit was used as the quantitative standard.

### 2.12 Macroscopic and histopathological analysis of intranasal inoculation site.

A limited postmortem examination focused on the upper and lower respiratory tract was conducted, with the aim of detecting any adverse reactions at the site of intranasal vaccine administration. Macroscopic pathological evaluation was performed in these regions, and any visible lesions were recorded. Whole retropharyngeal (left) lymph nodes (LN), tonsils and nasal turbinates of all animals were collected and fixed in 10%-buffered formalin to be evaluated by histopathology (hematoxylin-eosin and Ziehl-Neelsen staining). Retropharyngeal (right) LN of the animals from the BCG i.n. group were collected and stored at 4°C, for further processing for bacteriology.

Right retropharyngeal lymph nodes were processed and homogenized in distilled water. DNA extraction (ID Gene™ spin universal extraction kit, ID), real-time PCR (MTBC Duplex kit, ID), and mycobacterial culture in MGIT, Coletsos, and Löwenstein-Jensen media were performed as previously described (37). Multiplex PCR was performed to differentiate *M. bovis* wildtype from BCG, targeting the RD1 region (Rv3877/8) and the MPB70 gene (Rv2875), using the primer sets described previously (38).

### 2.13 Data analysis

Statistical analyses were conducted to assess differences between vaccination groups at each study timepoint, regarding IGRA, antibody levels, T-cell and AMs cytometry, and cytokine profile from BALF cells, using the non-parametric Kruskal-Wallis test, followed by a two-tailed uncorrected Dunn’s *post hoc* test. Differences between IFNγ responses against PPDB and DAC antigens were statistically analyzed using unpaired two-tailed Mann-Whitney test.

The relationship between CD4^+^IFNγ^+^ and CD8^+^IFNγ^+^ T-cell frequencies, as measured by flow cytometry, and IFNγ production by PBMCs, and correlation between activation and polarization markers in AMs, were examined using a two-tailed Spearman’s (ρ) correlation test.

## 3 Results

### 3.1 BCG elicited systemic IFNγ responses comparable to parenteral vaccination regimes

**Figure 2:**
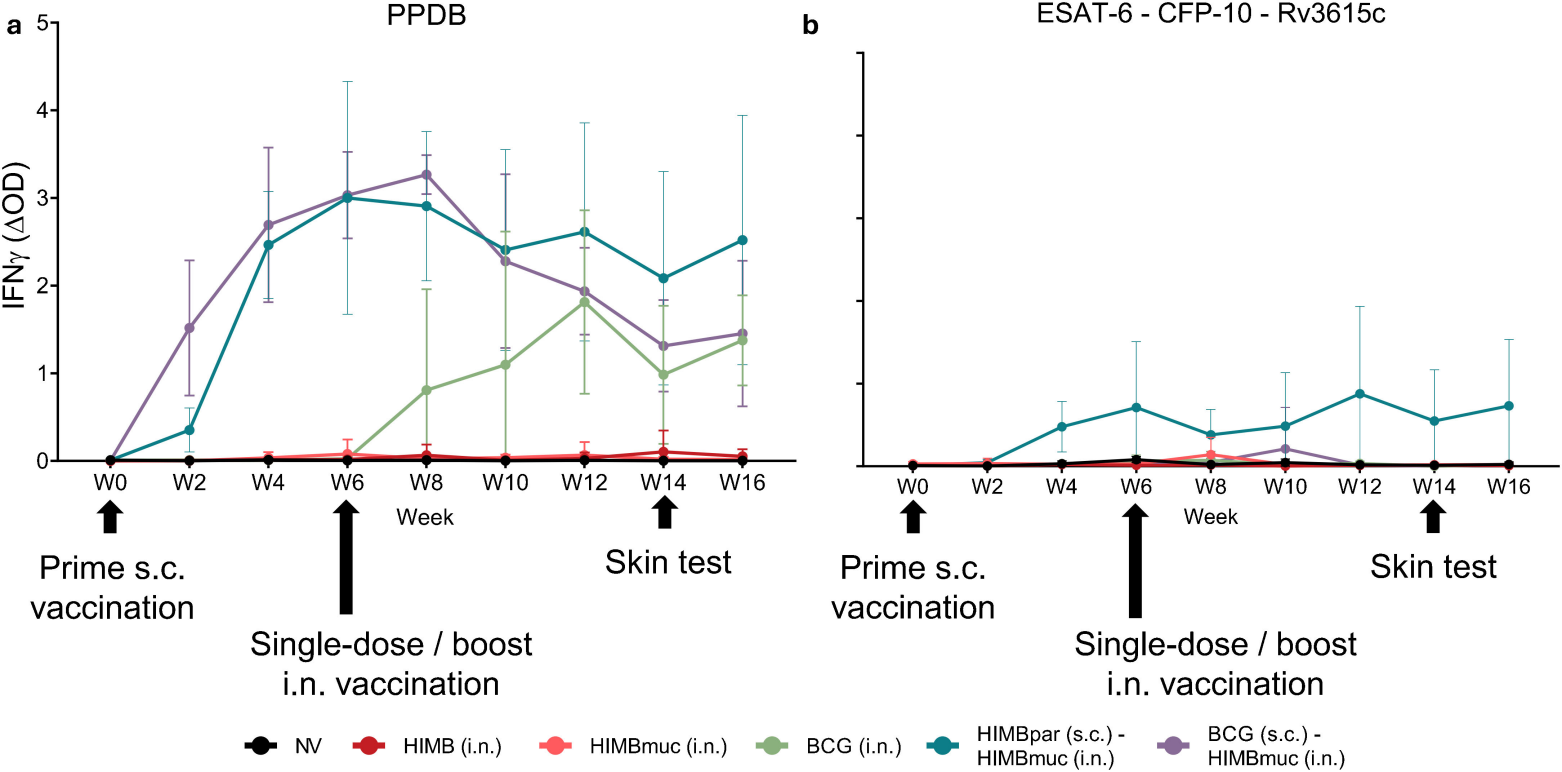
Whole-blood IFNγ responses. Mean **(a)** PPDB (*M. bovis* tuberculin) and **(b)** defined antigen cocktail (ESAT-6, CFP10 and Rv3615c)-specific IFNγ levels measured every two weeks throughout the study, measured by the IFNγ release assay (IGRA) with the ID Screen^®^ Ruminant IFNg kit (ID, Grabels, France). Results are expressed as mean Δ optical density (ΔOD) measured at 450 nm + Standard Deviation (SD). IFNγ levels were calculated as mean PPDB OD or defined antigen cocktail OD – PBS OD (ΔOD). Each color represents a different vaccination group. Comparison between vaccinated groups were made each week by non-parametric Kruskal-Wallis test with *post hoc* two-tailed Dunn’s test at each week; significant *p*-values are shown in **Supplementary Table S1**. Prime vaccination was administered subcutaneously at week 0 (HIMBpar s.c. or BCG s.c.), followed by intranasal boost (HIMBmuc i.n.) and single-dose intranasal (BCG i.n., HIMBmuc i.n., and HIMB i.n.) vaccination at week 6.

The mean IFNγ specific responses against PPDB and the defined antigens cocktail (DAC) for each treatment group are shown in **Figure 2**. PPDB-specific IFNγ responses gradually increased from the second week after prime s.c. vaccinations with HIMBpar and BCG, reaching peak levels at weeks 6 and 8, respectively. After week 8, IFNγ levels started to decline in the BCG (s.c.) – HIMBmuc (i.n.) group, while they remained steady in the HIMBpar (s.c.) – HIMBmuc (i.n.) group for the remainder of the study. After intranasal vaccinations administrated at week 6, IFNγ levels increased in the BCG i.n group, peaking at week 12, and reaching similar levels than BCG (s.c.) - HIMBmuc (i.n.) group. In contrast, the groups that received a single intranasal dose of HIMBmuc or HIMB did not elicit a clear PPDB-specific IFNγ response.

Regarding DAC-specific IFNγ responses, only the HIMBpar (s.c.) – HIMBmuc (i.n.) group showed detectable levels starting two weeks post-vaccination, which remained steady throughout the study (**Figure 2b**, significances shown in **Supplementary Table S2**). However, the responses induced by DAC in this group were consistently lower than the corresponding PPDB-specific responses at each time point after s.c. prime vaccination with HIMBpar (*p* < 0.05).

**Figure 3:**
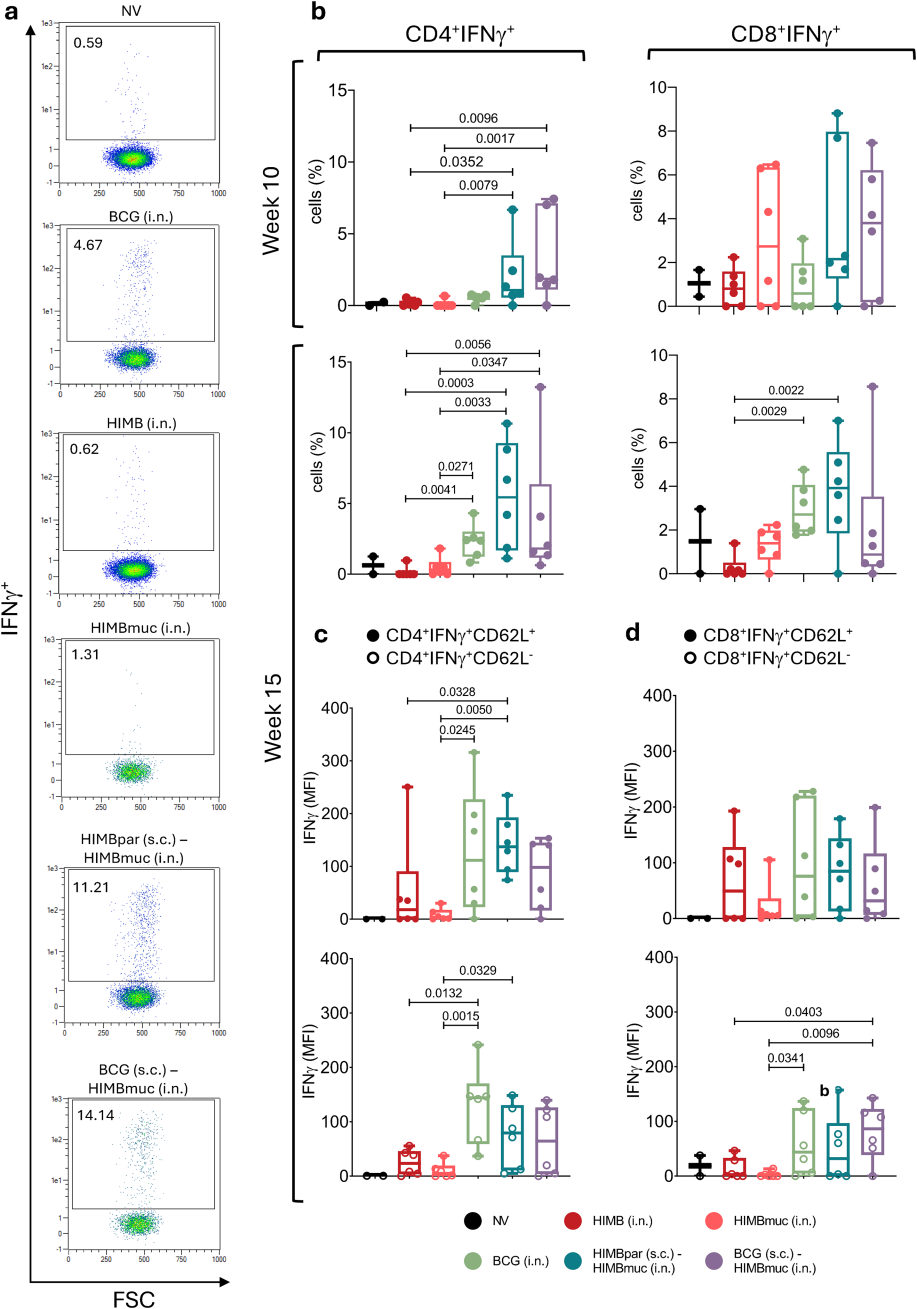
Flow cytometry assay: PPDB (*M. bovis* tuberculin)-specific T-cell subsets isolated from peripheral blood mononuclear cells (PBMCs). **(a)** Representative dot plots displaying the percentage of CD4^+^IFN γ^+^ T cells after PPDB stimulation. FSC, Forward Scatter **(b)** PPDB-specific CD4^+^IFN γ^+^ and CD8^+^IFN γ^+^ cell frequencies at week 10 and week 15 after s.c. vaccination (week 4 and week 9 after i.n. vaccination). Results are expressed as min. to max. % of cells from the CD4^+^ and CD8+ T-cell subsets. **(c, d)** Mean fluorescence intensity (MFI) of IFNγ produced by **(c)** CD4^+^IFN γ^+^ and **(d)** CD8^+^IFN γ^+^ Central Memory (solid pattern dots) and Effector (empty pattern dots) T-cell subsets at week 15 of the study. Results are expressed as min. to max. IFNγ MFI. Horizontal lines in every group represent the median values. Comparisons between vaccinated groups were made by non-parametric Kruskal-Wallis test with *post hoc* two-tailed Dunn’s test. Prime vaccination was administered subcutaneously at week 0 (HIMBpar s.c. or BCG s.c.), followed by intranasal boost (HIMBmuc i.n.) and single-dose intranasal (BCG i.n., HIMBmuc i.n., and HIMB i.n.) vaccination at week 6.

Flow cytometry assays from PBMCs showed that at week 10 (10 weeks after subcutaneous vaccinations and 4 weeks after intranasal vaccinations), PPDB-specific CD4^+^ IFNγ-producing cell percentages were significantly higher in the prime-boosted groups compared to the HIMB intranasally vaccinated groups (*p* < 0.05 compared with HIMBpar (s.c.) – HIMBmuc (i.n.), *p* < 0.01 compared with BCG (s.c.) – HIMBmuc (i.n.)), while CD8^+^ IFN γ^+^ cell subsets were also increased in some animals of these groups as well as the HIMBmuc i.n. group (**Figure 3b**). At week 15 (9 weeks after intranasal vaccinations), the frequency of PPDB-specific CD4^+^ increased in the BCG i.n. group, reaching levels comparable to both prime-boosted groups and significantly higher than those observed in the other intranasal single-vaccinated groups (*p* < 0.05 compared with HIMB i.n., *p* < 0.01 compared with HIMBmuc i.n.). Frequencies of CD8^+^ IFN γ^+^ at this time point were also significantly higher in the HIMBpar (s.c.) - HIMBmuc (i.n.) and BCG i.n. groups compared to HIMB intranasally vaccinated animals (*p* < 0.01).

Further analysis of PPDB-specific CD4^+^ T-cell subsets at week 15, revealed that the BCG i.n., HIMBpar (s.c.) -HIMBmuc (i.n.) and BCG (s.c.) - HIMBmuc (i.n.) groups exhibited higher mean fluorescence intensity (MFI) of IFNγ in both effector (CD62L^-^) and central memory (CD62L^+^) CD4^+^ and CD8^+^ IFNγ-producing T-cells subsets, compared to the HIMB i.n. groups and non-vaccinated animals (**Figures 3c, d**).

When assessing the relationship between IFNγ production (measured by ELISA) and T-cell subset frequencies (**Supplementary Figures S4a, b**), a stronger correlation of IFNγ levels with CD4^+^ IFNγ^+^ cells (Spearman ρ = 0.8898, *p* < 0.0001) than with CD8^+^IFNγ^+^ cells (Spearman ρ = 0.5019, *p* < 0.01) at week 15 was observed. Furthermore, the correlation between T-cell percentages and IFNγ production was stronger at week 15 than at week 10 for both CD4^+^ IFNγ^+^ (Spearman ρ = 0.6211, *p* < 0.0001) and CD8^+^ IFNγ^+^ T-cell subsets (Spearman ρ = 0.3266, *p* > 0.05).

### 3.2 Parenteral HIMB administration induced stronger IgG responses compared to other vaccines

**Figure 4:**
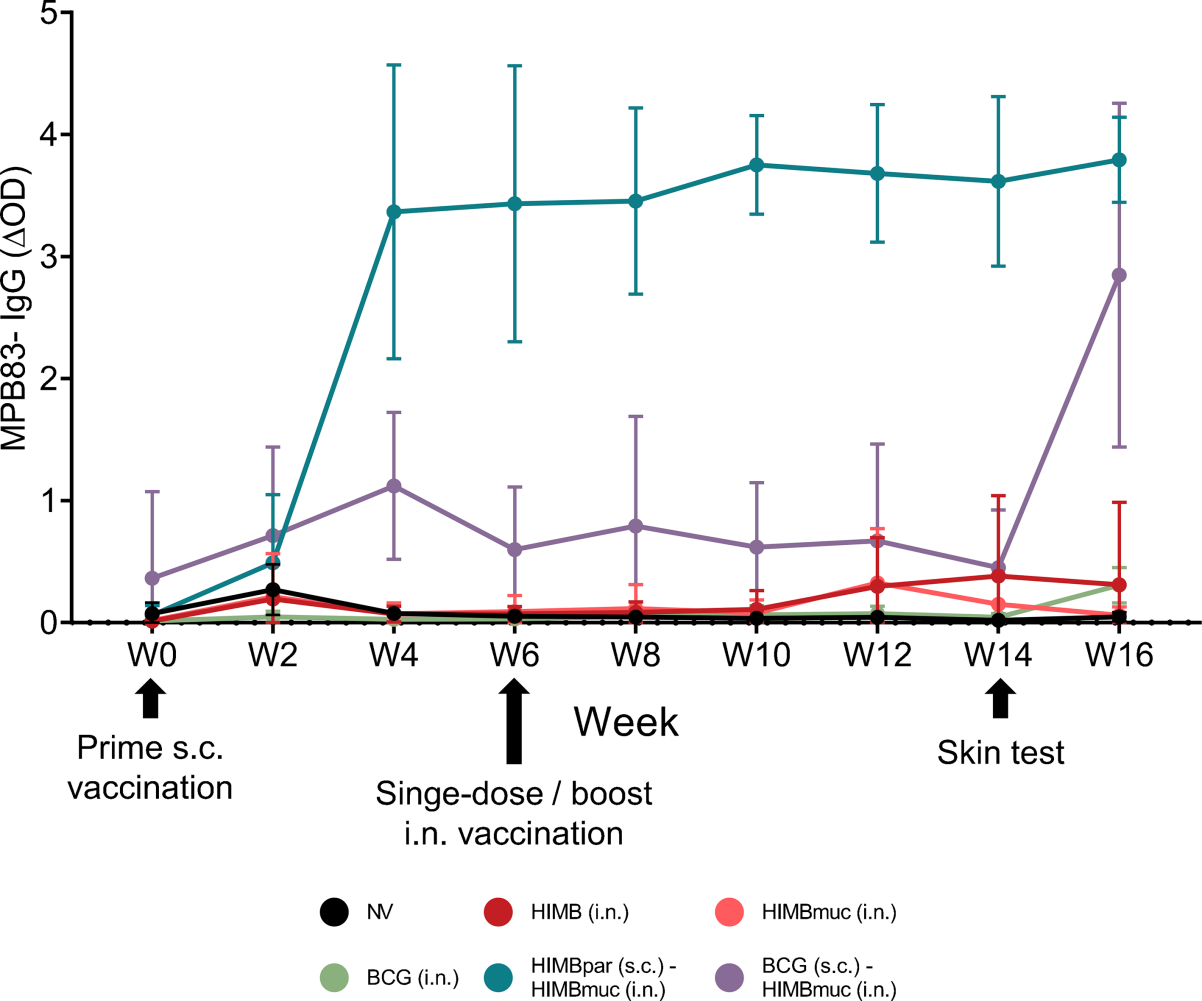
Serum MTBC-specific IgG responses. MPB83-specific IgG levels measured by indirect ELISA every two weeks throughout the study. At week 14 skin test was performed. Results are expressed as mean Δ optical density (ΔOD) measured at 450 nm + Standard Deviation (SD). MPB83-IgG levels were calculated as mean OD of antigen-coated well – OD of non-coated well (ΔOD). Each color represents a different vaccination group. Comparison between vaccinated groups were made each week by non-parametric Kruskal-Wallis test with *post hoc* two-tailed Dunn’s test at each week; significant *p*-values are shown in **Supplementary Table S2**. Prime vaccination was administered subcutaneously at week 0 (HIMBpar s.c. or BCG s.c.), followed by intranasal boost (HIMBmuc i.n.) and single-dose intranasal (BCG i.n., HIMBmuc i.n., and HIMB i.n.) vaccination at week 6.

The kinetics of serum IgG to MPB83 antigen throughout the study is shown in **Figure 4** (significances shown in **Supplementary Table S3**). After prime s.c. vaccination with BCG and HIMBpar, MTBC-specific IgG levels started to increase. From week 4 onwards, mean IgG levels induced by HIMBpar were higher than those induced by BCG, and differences persisted unchanged after HIMBmuc i.n. boosting of both groups performed at week 6. Both groups maintained steady IgG levels throughout the study. In contrast, the single-dose intranasal vaccination groups did not show detectable IgG levels until week 12, when a mild increase was observed in both HIMB i.n groups. The skin test conducted at week 14 triggered an enhanced antibody response by week 16 in the BCG (s.c.) - HIMBmuc (i.n.) group and, to a lesser extent, in the BCG i.n. group. Antibody levels in the other groups remained largely unchanged after the skin test.

### 3.3 BCG i.n. and parenteral vaccination regimes induced proinflammatory lung mucosal immunity

**Figure 5:**
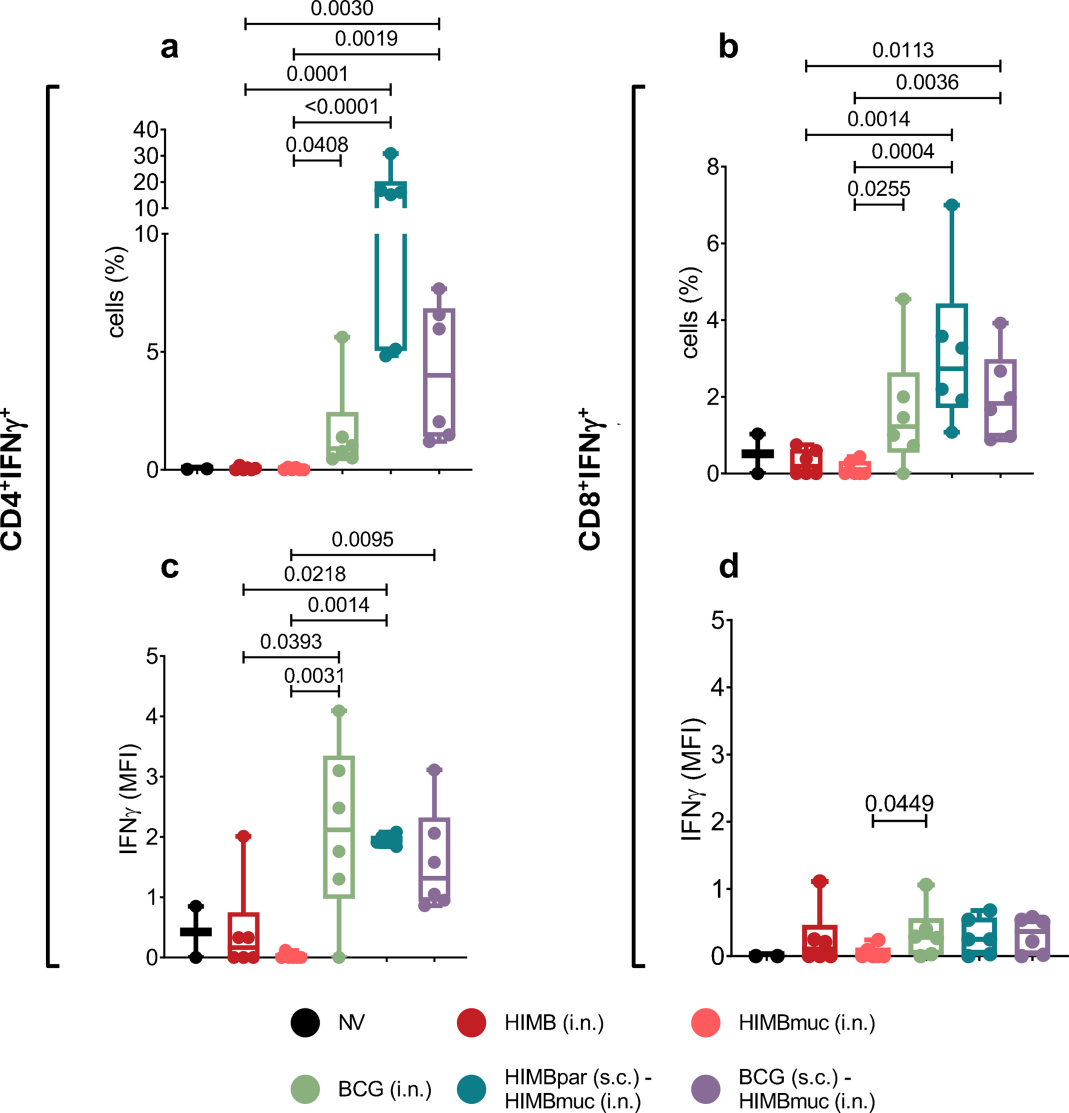
Flow cytometry assay: PPDB (*M. bovis* tuberculin)-specific T-cell subsets isolated from bronchoalveolar lavage fluid (BALF). PPDB-specific **(a)** CD4^+^IFN γ^+^ and **(b)** CD8^+^IFN γ^+^ cell frequencies at week 16 after s.c. vaccination (week 10 after i.n. vaccination). Mean fluorescence intensity (MFI) of IFNγ produced by **(c)** CD4^+^IFN γ^+^ and **(d)** CD4^+^IFN γ^+^ T cell subsets. Results are expressed as min. to max. IFNγ MFI. Horizontal lines in every group represent the median values. Comparison between vaccinated groups were made each week by non-parametric Kruskal-Wallis test with *post hoc* two-tailed Dunn’s test. Prime vaccination was administered subcutaneously at week 0 (HIMBpar s.c. or BCG s.c.), followed by intranasal boost (HIMBmuc i.n.) and single-dose intranasal (BCG i.n., HIMBmuc i.n., and HIMB i.n.) vaccination at week 6.

The prime-boosted groups showed significantly higher frequencies of antigen-specific CD4^+^ IFN γ^+^ and CD8^+^ IFN γ^+^ T-cells in BALF compared to both single-dose HIMB intranasally vaccinated groups (*p* < 0.001 and *p* < 0.05, respectively, **Figures 5a, b**), and they were also significantly higher on the BCG i.n. group compared to HIMBmuc i.n. group (*p* < 0.05). Moreover, the production of IFNγ, measured by MFI, within CD4^+^ IFN γ^+^ T-cells was higher in the BCG i.n. and prime-boosted groups compared to the single-dose intranasal HIMB groups (**Figure 5c**). This difference was statistically significant when compared to the HIMBmuc i.n. group (*p* < 0.01), and between BCG i.n. and HIMBpar (s.c.) - HIMBmuc (i.n.) groups and HIMB i.n. without adjuvant (*p* < 0.05). The IFNγ production of CD8^+^ IFN γ^+^ T-cells from the BCG i.n. group was also significantly higher than CD8^+^ IFN γ^+^ T-cells from HIMB i.n. vaccinated animals (*p* < 0.05).

**Figure 6:**
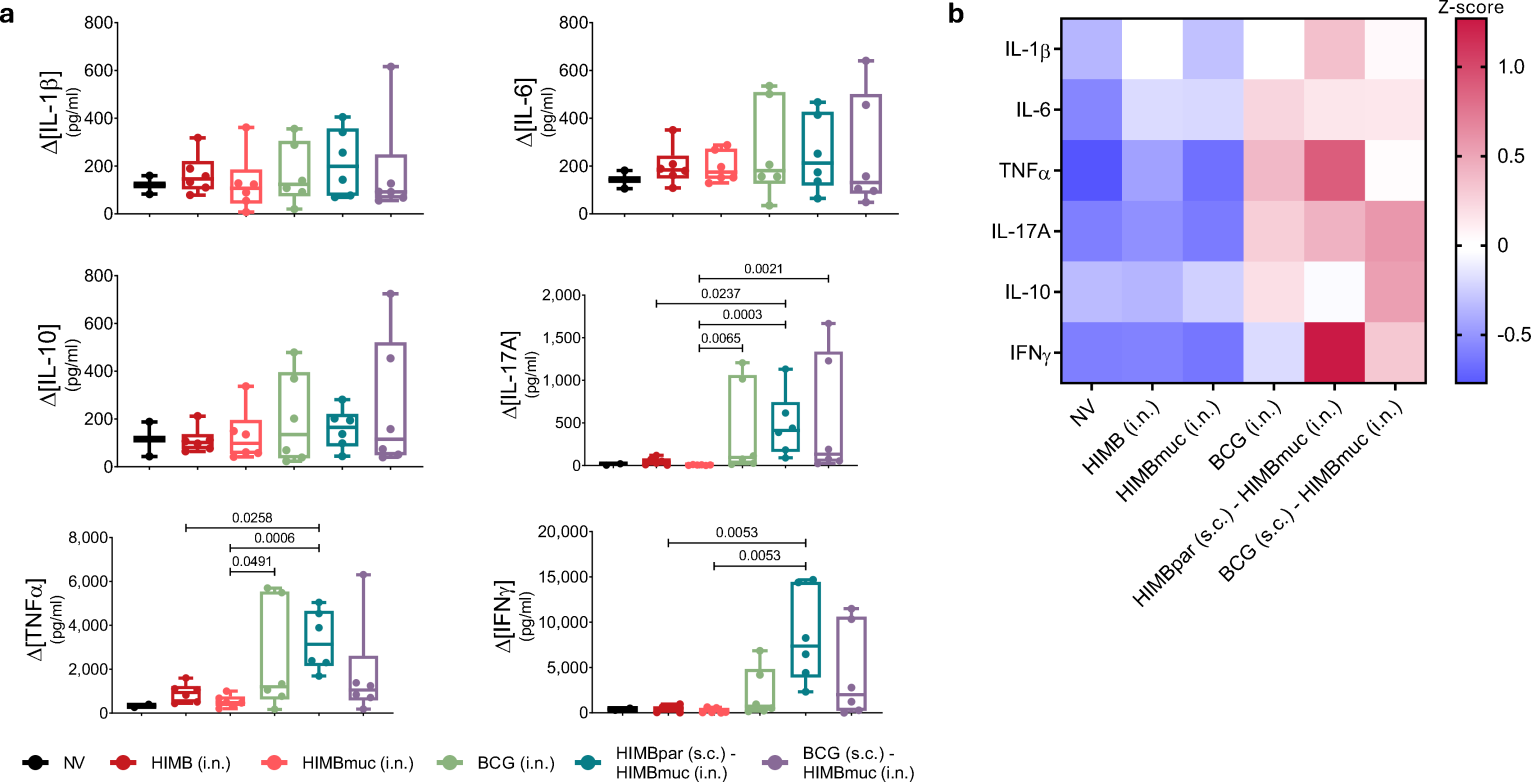
Cytokine profile produced by cells isolated from bronchoalveolar lavage fluid (BALF), 16 weeks after s.c. vaccination (10 weeks after i.n. vaccination). **(a)** Cytokine production by cells obtained from BALF stimulated with HIMB and RPMI as background control. HIMB-specific cytokine levels were calculated subtracting RPMI background levels to HIIMB-stimulated samples. Comparison between vaccinated groups were made by non-parametric Kruskal-Wallis test with *post hoc* two-tailed Dunn’s test. **(b)** Heat-map represents z-score normalization for each cytokine to account for differences in scale between cytokines. The color gradient represents the magnitude of response, red indicates a higher-than-average response, while blue represents a lower-than-average response within each cytokine. Prime vaccination was administered subcutaneously at week 0 (HIMBpar s.c. or BCG s.c.), followed by intranasal boost (HIMBmuc i.n.) and single-dose intranasal (BCG i.n., HIMBmuc i.n., and HIMB i.n.) vaccination at week 6.

Regarding the antigen-specific cytokine production analysis in BALF cells, stimulation with HIMB led to increased production of all cytokines across all groups compared to non-stimulated samples (**Figure 6a**). However, the HIMB-specific levels of all cytokines, particularly IL-17A, TNFα and IFNγ, were significantly higher in the BCG i.n., HIMBpar (s.c.) - HIMBmuc (i.n.), and BCG (s.c.) - HIMBmuc (i.n.) groups compared to the intranasal HIMB-only vaccinated groups (**Figures 6a, b**).

### 3.4 Vaccine-induced activation and proinflammatory polarization of AMs

**Figure 7:**
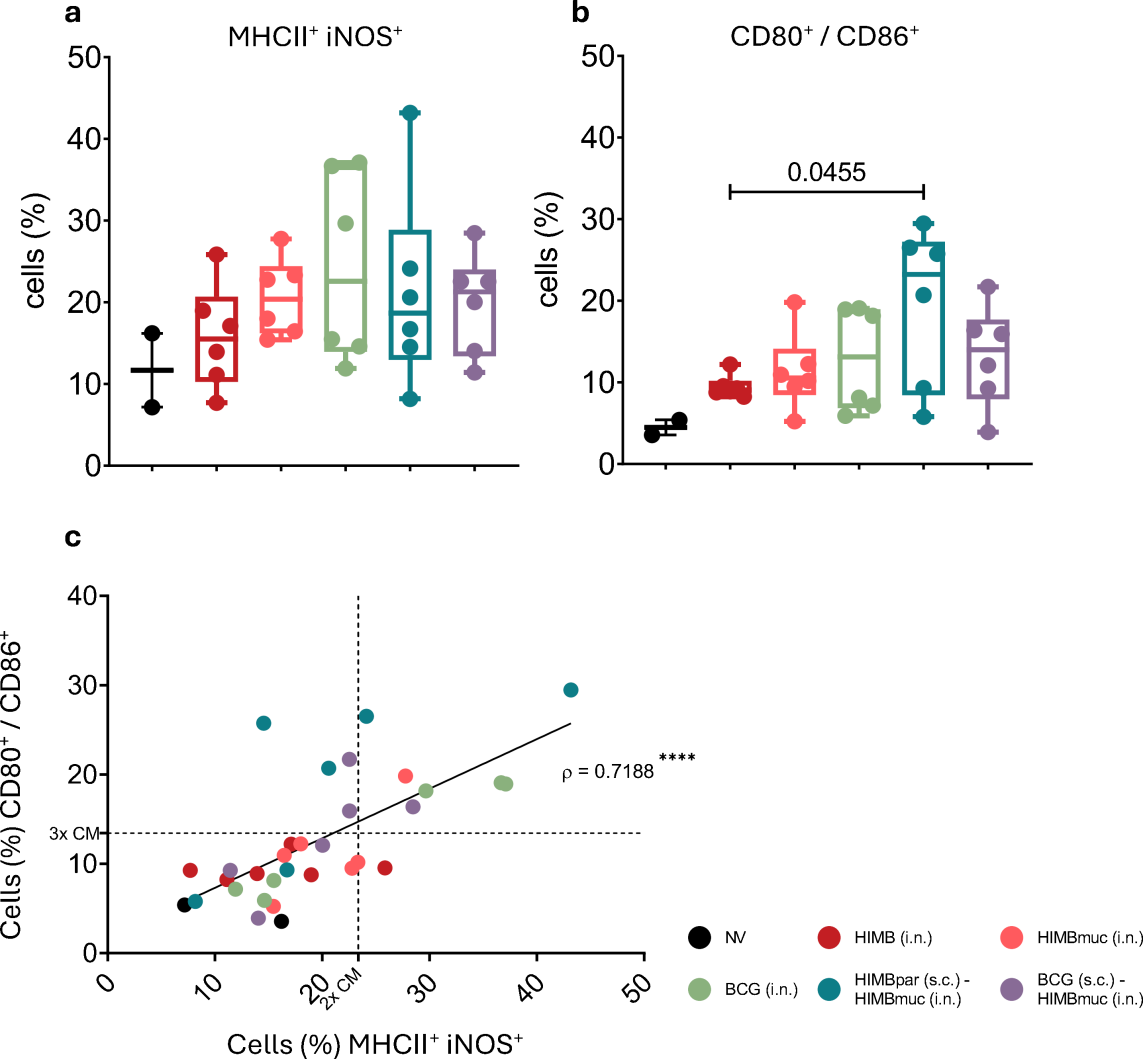
Alveolar macrophage (AMs) subsets isolated from bronchoalveolar lavage fluid (BALF) at week 16 after s.c. vaccination (week 10 after i.n. vaccination). **(a)** MHCII^+^ iNOS^+^ AMs frequencies. **(b)** CD80^+^ and/or CD86^+^ AMs frequencies. Results are expressed as min. to max. % of cells from the alive cell subsets, horizontal lines in every group represent the median values. **(c)** Correlation of MHCII^+^ iNOS^+^ AMs frequencies with CD80^+^ and/or CD86^+^ AMs frequencies. Each color represents a different vaccination group, each dot represents an individual. Dashed lines represent responder thresholds: X-axis (vertical) = 2× mean of unvaccinated controls (CM); Y-axis (horizontal) = 3× mean of unvaccinated controls (CM). Individuals in the upper-right quadrant are considered high responders. Comparison between vaccinated groups were made by non-parametric Kruskal-Wallis test with *post hoc* one-tailed Dunn’s test; **** *p* < 0.0001 (Two-tailed Spearman (ρ)). Prime vaccination was administered subcutaneously at week 0 (HIMBpar s.c. or BCG s.c.), followed by intranasal boost (HIMBmuc i.n.) and single-dose intranasal (BCG i.n., HIMBmuc i.n., and HIMB i.n.) vaccination at week 6.

AMs were considered activated when positive for both Major Histocompatibility Complex-II (MHCII) and iNOS, and M1-polarized when positive for CD80 and/or CD86. All vaccinated groups showed an increased frequency of activated (**Figure 7a**) and proinflammatory (M1)-polarized (**Figure 7b**) AMs phenotypes compared to non-vaccinated animals.

Notably, certain individuals in the BCG i.n., HIMBpar (s.c.) - HIMBmuc (i.n.), and BCG (s.c.) - HIMBmuc (i.n.) groups exhibited a consistently higher frequency of both activated and M1-polarized cells, and a strong correlation (Spearman ρ = 0.7188, *p* < 0.0001) was found between the individual frequencies of activated and polarized cells (**Figure 7c**).

### 3.5 Intranasal inoculation safety data

No macro nor microscopic lesions were observed in the nasal cavity, tonsils or left retropharyngeal lymph nodes of any of the animals. Except for a single animal from the BCG i.n. group which presented a small focal granulomatous lesion with multinucleated giant cells (Langhans cells) in the left retropharyngeal lymph node (**Supplementary Figures S5a, b**). MTBC was isolated from liquid culture (BBL MGIT) from the contralateral lymph node of this one and another animal from the same group. BCG was detected in these samples by PCR. Ziehl-Neelsen staining did not reveal the presence of acid-fast bacilli in any of the analyzed sections.

## 4 Discussion

Developing vaccines against MTBC which can elicit an effective mucosal immunity is critical for enhancing early immune responses at the primary site of infection (39, 40). Intranasal (i.n.) delivery platforms offer a promising strategy to elicit both local and systemic immunity, potentially improving upon the limited and variable protection conferred by conventional parenteral BCG and HIMB vaccination, particularly against pulmonary disease (41, 42) which is the most frequent clinical form of active TB in humans as well as in livestock (1, 43, 44). Building on this concept, revaccination represents another strategy to enhance the limited protection conferred by subcutaneous vaccination. However, boosting through the same route as the prime has repeatedly shown little to no additional benefit (45).

In contrast, intranasal delivery as booster may more effectively amplify the pre-existing immunity induced by the initial parenteral vaccination, activating innate and adaptive immunity at a local level unlikely to be achieve by purely parenteral administration. Although BCG is one of the most widely used vaccines globally, and has a well-established safety profile in immunocompetent individuals, a heat-inactivated vaccine could provide several advantages over a live-replicating formulation: improved safety profile, including immunocompromised host; as well as greater stability, which may facilitate storage, distribution and regulatory approval. In previous studies, immunogenicity and efficacy of parenteral HIMB has been investigated in goat and sheep (13, 14, 16). Given the emerging interest in mucosal vaccination—particularly the promising results obtained with intranasal BCG—it is also of consideration to assess HIMB delivered via the same intranasal route.

The lack of animal models that accurately replicate the complex immune responses observed in humans following either *M. tuberculosis* infection or BCG vaccination hinders the preclinical evaluation and prediction of vaccine efficacy (46, 47). Over the past fifteen years, several studies have proposed the goat model of TB as a feasible and translational platform to resemble key immunopathological features of active human TB (48–50), as well as the immune responses induced by BCG and other mycobacterial vaccines (7, 35, 51, 52). Goats are a suitable TB model as they develop early pulmonary lesions, necrotic granulomas, and disease progression similar to humans and cattle, and they mount comparable cellular and innate immune responses. They also offer practical advantages over cattle, being smaller, easier to handle, more cost-effective, and better adapted to BSL-3 studies. The relevance of this model also relies on the fact that goats are natural hosts of tuberculosis (53), thereby providing not only a robust platform for translational research but also direct applicability of the findings to the target host species.

In this study, we demonstrate that vaccination of goat kids with a single dose of intranasal BCG elicited systemic specific cell-mediated proinflammatory immune responses comparable to those induced by parenteral vaccination with BCG and HIMB (13, 54). Intranasal BCG was able to induce stronger MTBC-specific production of IFNγ and increase the frequencies of CD4^+^IFN γ^+^ and CD8^+^IFNγ T-cells in peripheral blood than intranasal HIMB vaccines at nine weeks after vaccination, regardless of the use of a mucosal adjuvant, suggesting that mucosal delivery of BCG can trigger robust systemic Th1 immune responses. This is consistent with previous studies in non-human primate models where intradermal and pulmonary BCG vaccination induced similar peripheral CD4^+^ T-cell proliferation and IFN-γ responses (55), and aerosol BCG vaccination increased the frequency of systemic PPD-specific IFNγ–secreting cells and elicited Th1- and Th17-type cytokine responses in both CD4^+^ and CD8^+^ T-cells from PBMCs (31, 56). The enhanced systemic responses elicited by intranasal BCG compared to intranasal HIMB may be explained by the fact that BCG is a live attenuated vaccine with replication capacity (57). This allows BCG not only to act at the mucosal surface but also to drain to regional lymph nodes, as supported by histopathological and bacteriological findings. Similarly, prime-boosted groups showed higher frequencies of antigen-specific CD4^+^ and CD8^+^ T-cells producing IFNγ compared to the HIMB-only and non-vaccinated groups.

Antigen-specific IFNγ responses increased notably after the subcutaneous prime and intranasal BCG vaccinations, confirming effective induction of cell-mediated immunity. HIMBpar (s.c.) - HIMBmuc (i.n.)As expected, IFNγ production peaked after s.c. HIMB and BCG vaccination (13). Although overall IFNγ levels were broadly comparable among these vaccinated groups, the HIMBpar (s.c.) – HIMBmuc (i.n.) regimen showed a more sustained response and was the only one to elicit detectable IFNγ against ESAT-6/CFP-10/EspC, suggesting enhanced antigen-specific engagement. In the BCG (s.c.) - HIMBmuc (i.n.) group, however, IFNγ levels gradually declined, consistent with previous findings (7, 57), suggesting that a heterologous boost using HIMBmuc i.n. after subcutaneous BCG does not seem to have an improved effect on IFNγ production. HIMBpar (s.c.) - HIMBmuc (i.n.). By comparison, animals vaccinated with intranasal HIMB alone or with adjuvant, as well as non-vaccinated animals, failed to elicit detectable IFNγ responses, highlighting the limited immunogenicity of HIMB alone when delivered intranasally.

In general, systemic immune responses induced by vaccination over time showed a slight decline in the BCG (s.c.) - HIMBmuc (i.n.)–vaccinated group, both in IFNγ release and in the proportion of IFNγ–producing CD4+ and CD8+ T-cells. In contrast, the responses were maintained or even increased in the HIMBpar (s.c.) - HIMBmuc (i.n.)–vaccinated group. The intranasal BCG group exhibited a marked increase between the two sampling points, likely reflecting that the peak IFNγ response had not yet been reached. Additionally, antigen-specific IFNγ production by PBMCs measured by ELISA showed better direct correlation with CD4^+^ IFN γ^+^ T-cell frequencies than with CD8^+^ T-cell subsets (see **Supplementary Figures S4a, b**), indicating that IFNγ production is primarily driven by CD4^+^ T-cells, consistent with previous reports (58).

Long lasting protection of effective TB vaccines must relies on durable T-cell memory subsets, such as central memory T-cells (T_CM_), which exhibit high proliferative potential, and effector memory T-cells (T_EM_), which display robust cytokine secretion capacity (59, 60). When memory CD4^+^ and CD8^+^ T-cell subsets were further characterized using CD62L as memory marker, both antigen-specific IFNγ-producing central memory T-cells (CD62L^+^) and effector T-cells (CD62L⁻) from BCG i.n. and prime-boosted groups produced higher levels of IFNγ than memory and effector cells isolated from non-vaccinated and HIMB i.n. vaccinated individuals. These findings are consistent with previous studies reporting robust proliferation of memory PPDB-specific CD4+ T-cells (CD4^+^CD45RO^+^) in cattle (61) and goats (7) vaccinated and revaccinated with s.c. BCG, respectively; and with Melgarejo et al. (2022), who reported that frequencies of PPDB-specific IFNγ-producing memory T-cells were positively associated with favorable disease outcome in HIMBpar-vaccinated goats.

Even though antibody responses are not considered essential to protective immunity against MTBC, vaccine-induced seroconversion and MTBC-specific IgG levels serve as indicators of vaccine responsiveness at both the group and individual levels (34) It is well established that s.c. HIMB vaccination elicits an earlier and more robust humoral immune response compared to s.c. BCG administration (13, 62). In our study, only HIMBpar (s.c.) - HIMBmuc (i.n.) and BCG (s.c.) - HIMBmuc (i.n.) groups induced detectable levels of antibodies in blood, considerably higher in HIMBpar (s.c.) - HIMBmuc (i.n.) group. These results may reflect that MPB83 is constitutively expressed in virulent *M. bovis*, whereas BCG strains produce it only at very low levels (63). Additionally, both BCG vaccinated groups, whether intranasally or subcutaneously, showed a boost in antibody levels at week 16 of the study, after skin test was performed, markedly larger in the s.c. BCG (mean ΔOD goes from 0.451 at week 14 to 2.848 at week 16) than the i.n. BCG (mean ΔOD goes from 0.044 at week 14 to 0.307 at week 16) group as previously described (12, 34). This boosting phenomenon of increased MTBC-specific IgG levels has been well characterized before (63). While HIMB intranasally vaccinated groups did not show increment in antibody responses, aligning with the results observed for cell-mediate immunity.

It has been previously shown that mucosal vaccination with BCG (27, 64) and an attenuated *M. tuberculosis* vaccine (65) can induce local T-cells responses in the respiratory system, and have the ability to provide protection against pulmonary infection. In line with these finding, our study shows that, in addition to systemic responses, BCG i.n. also induced robust proinflammatory immune responses at the lung mucosa, the primary site of infection of MTBC (43). This group exhibited elevated levels of MTBC-specific proinflammatory cytokine production and increased frequencies of T-cell subsets in BALF, as well as polarization of AMs toward a proinflammatory M1 phenotype and increased activation state.

While it is hypothesized that parenteral vaccination may not effectively prime antigen-specific T-cells in the lung mucosa, thus limiting local protection, existing literature presents mixed results. Some studies suggest limited mucosal T-cell recruitment following systemic vaccination (66, 67), while others indicate that parenteral vaccination can still promote mucosal immune responses, including the induction of memory macrophages and trained immunity (68), antigen-specific tissue-resident CD4^+^ T-cell population (69, 70), and mucosal multifunctional T_EM_ cells in the lung (71). Following this line of evidence, in this study we observed that prime-boosted groups presented higher frequencies of antigen-specific CD4^+^ and CD8^+^ IFNγ producing T-cells isolated from BALF than HIMB-only vaccinated animals and non-vaccinated animals. Moreover, this CD4^+^IFNγ lymphocyte subsets isolated from BALF from BCG i.n. and prime-boosted groups produced higher levels of IFNγ than those from the rest of the groups. This shows that parenteral vaccines were capable of recruiting antigen-specific IFNγ producing T-cells to the lung, in a similar manner to a single-dose of intranasal BCG, while a single-dose or boost with HIMB does not impact lung recruitment of T-cells.

BCG i.n., HIMBpar (s.c.) - HIMBmuc (i.n.) and BCG (s.c.) - HIMBmuc (i.n.) groups showed higher antigen-specific proinflammatory cytokine production in BALF, than HIMB intranasally vaccinated groups, when restimulated *in vitro* with HIMB. Overall, *ex vivo* HIMB stimulation of BALF cells led to increased production of IL-1β, IL-6, TNFα, and IL-10 across all groups indicating that it induces the production of these cytokines in BALF cells regardless of vaccination status. In contrast, IL-17A and IFNγ were produced following antigen re-exposure *ex vivo*, as non-vaccinated and HIMB intranasally vaccinated animals did not produce significant levels of these cytokines. TNFα followed a mixed pattern, showing a slight increase upon *ex vivo* HIMB stimulation regardless of vaccination status, but a more pronounced, specific increase in BCG i.n. and prime boosted groups. IL-10, an anti-inflammatory cytokine, was also elevated, possibly as a regulatory mechanism to control excessive inflammation.

While most TB vaccine research over the past decades has focused on the adaptive immune response, increasing evidence suggests that innate immune mechanisms within the lung mucosa—particularly AMs, which are the first immune cells to encounter MTBC and serve as its primary host (72, 73) —may play a crucial role in the early control of infection, even before the activation of T-cell responses (32, 33, 74). Classically activated macrophages (M1) are known to have a restrictive phenotype for growth of the bacilli (75, 76), contributing to the control of mycobacterial replication. In our study, all vaccinated groups displayed elevated frequencies of markers for both activated and proinflammatory (M1-polarized) AMs phenotypes compared to non-vaccinated controls, suggesting enhanced innate immune activation induced by vaccination. M1 macrophages are characterized by the production of pro-inflammatory cytokines, their high antigen-presenting capacity, upregulation of co-stimulatory molecules involved in T-cell activation, and increased production of reactive nitrogen and oxygen species, which contribute to the macrophage’s antimicrobial activity (77–79). Here, the results indicated that vaccination promoted all these M1-associated functions, as evidenced by increased production of IL-1β, IL-6 and TNFα, elevated expression of MHC II, CD80 and CD86, and upregulation of iNOS. These findings suggest that vaccination induced a shift toward a more pro-inflammatory, bactericidal macrophage phenotype. This effect was most pronounced in the prime-boosted groups and the intranasal BCG group, where the strongest macrophage activation and M1 polarization was observed. Consistent with previous studies (68, 80–82), these results highlight the potential of mucosal and parenteral BCG vaccination to activate AMs and enhance early immune responses at the site of MTBC entry. Moreover, there was a strong positive correlation between activation and M1 markers at the individual animal level—suggesting that most activated macrophages had also adopted the M1 phenotype.

Regarding the use of the mucosal adjuvant in intranasal vaccination with heat-inactivated *M. bovis*, our results show no clear differences between the two intranasally vaccinated HIMB groups, suggesting that the adjuvant does not provide a marked benefit under the study conditions. However, a slight increase in CD8^+^ T-cell responses and AMs activation was observed in the HIMBmuc i.n. group compared to HIMB intranasal alone. This may suggest that a different adjuvant dose or repeated administration could be necessary to reveal more pronounced immunogenic effects of the adjuvant. In addition, HIMBmuc i.n. may have contributed to maintaining steady IFNγ levels in the HIMBpar (s.c.) - HIMBmuc (i.n.) group, potentially acting as a boost to the parenteral prime. Nonetheless, the current study design does not allow for definitive conclusions regarding the adjuvant’s boosting capacity and additional studies evaluating increased doses or repeated intranasal administrations of HIMB, with or without adjuvant, are needed.

In conclusion, intranasal BCG vaccination demonstrated strong potential as a mucosal immunization strategy against TB, effectively inducing both systemic and, notably, robust local immune responses in the lung. BCG i.n. elicited systemic antigen-specific cellular immunity comparable to that induced by parenteral BCG and HIMB vaccination. Importantly, BCG i.n. and prime-boost approaches triggered robust pro-inflammatory, antigen-specific responses within the lung mucosa, highlighting its capacity to target the primary site of infection. These results support further investigation into the efficacy of respiratory mucosal BCG vaccination to prevent spreading of early MTBC infection.

## Data Availability

The raw data supporting the conclusions of this article will be made available by the authors, without undue reservation.
