# Supplementary material for: Intranasal BCG vaccination induces systemic and pulmonary mucosal immune responses against tuberculosis in a goat model
Source: Front Immunol. 2026 Jan 2;16:1740197. doi: 10.3389/fimmu.2025.1740197 (PMC12808410; doi:10.3389/fimmu.2025.1740197)
Supplement: Supplementary file 1 [file Table1.docx]

**Supplementary Materials**

**
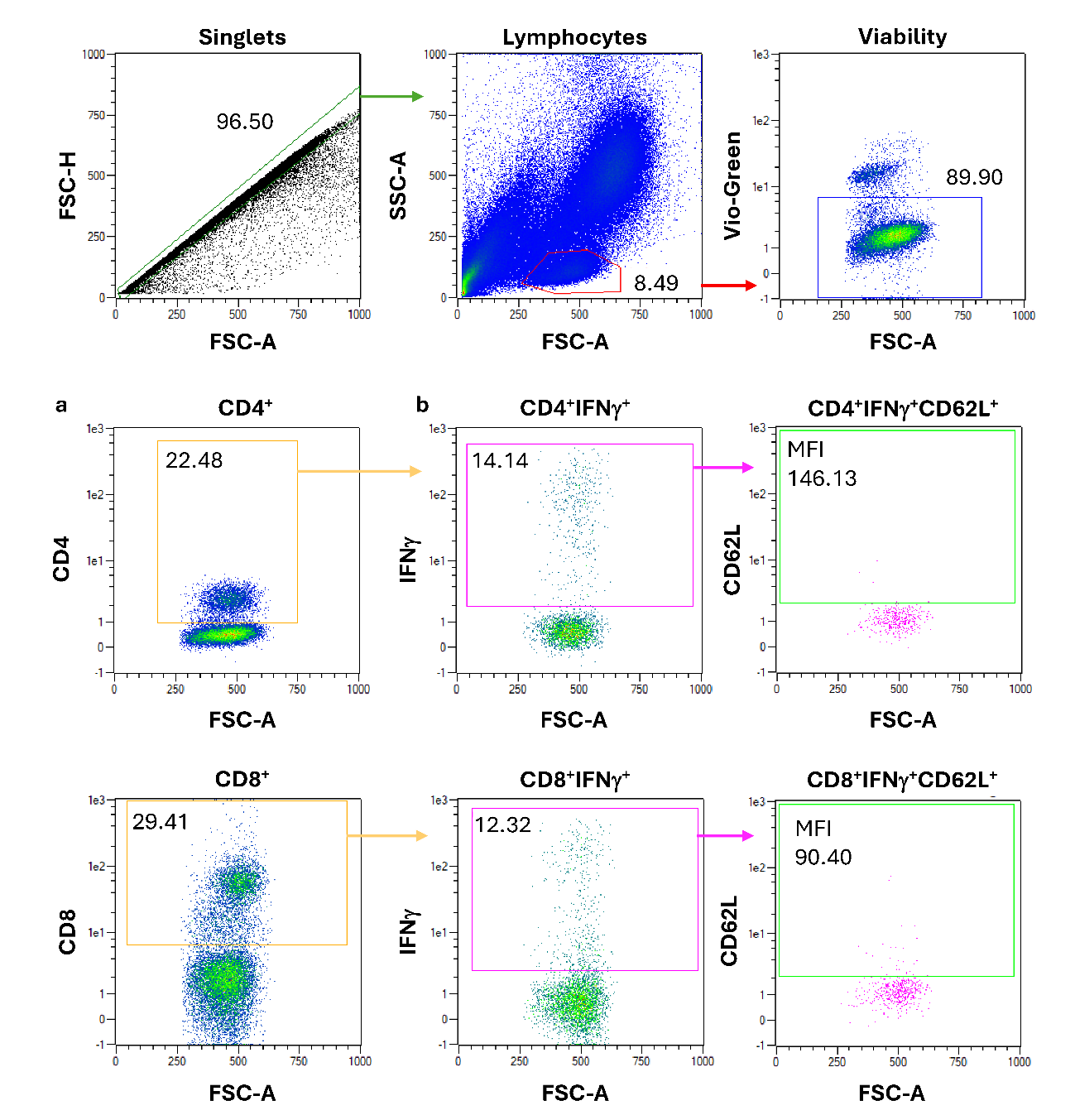
**

**b**

**a**

**Figure S1. Gating strategy used to identify IFNγ⁺ T-cell populations in PPDB-stimulated and unstimulated peripheral blood mononuclear cells (PBMCs) collected at week 15 post-s.c. vaccination (week 9 post-i.n. vaccination).** The gating strategy shown corresponds to animal 370, vaccinated using the BCG-HIMBmuc prime-boost strategy. (**a**) Frequencies of CD4⁺IFNγ⁺ and CD8⁺IFNγ⁺ T-cells in the PPDB-stimulated samples. (**b**) Mean fluorescence intensity (MFI) from CD4+IFNγ+ and CD8+IFNγ+ memory T-cell (CD62L+) from PPDB-stimulated samples.

**
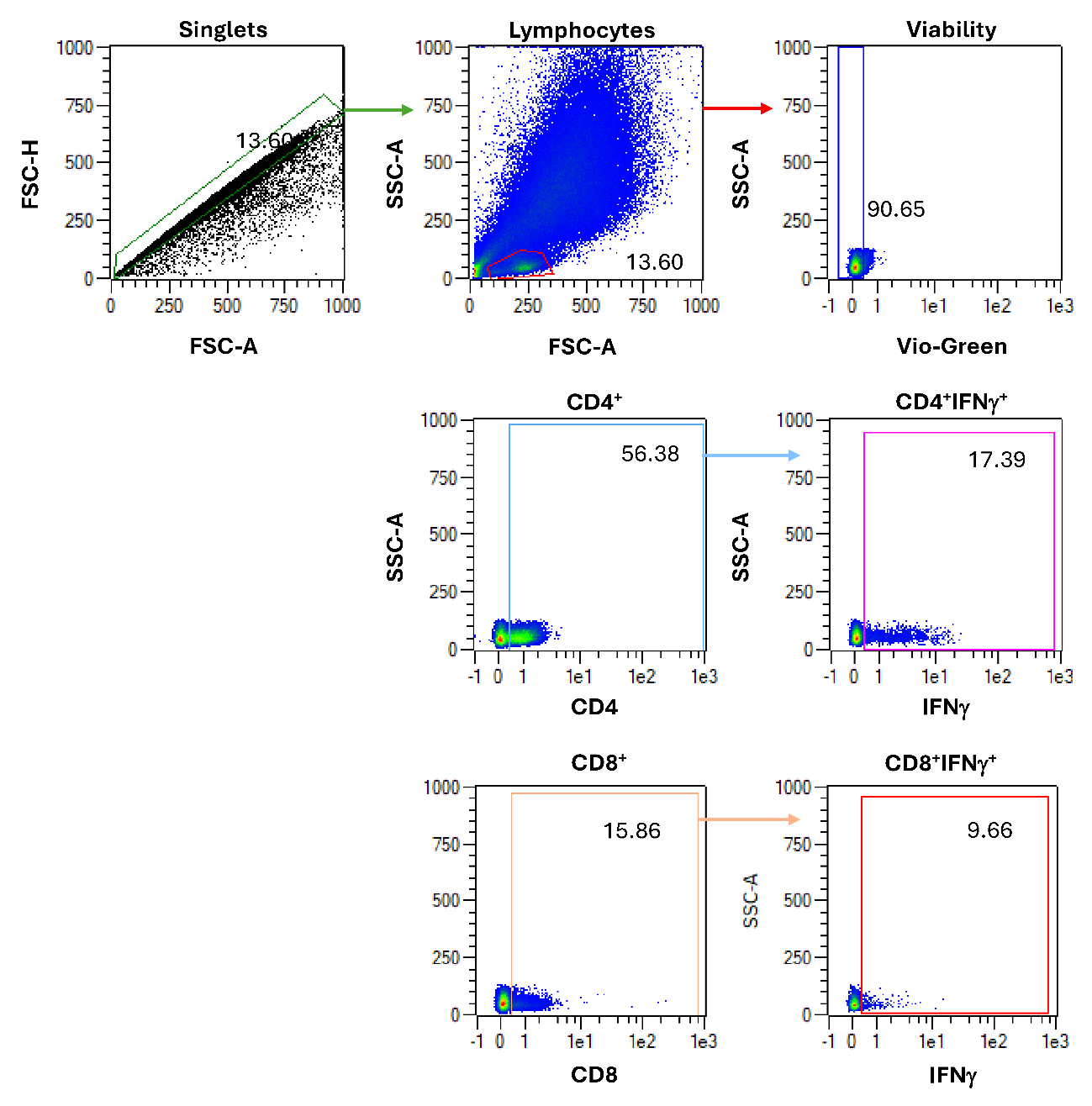
**

**Figure S2. Gating strategy used to identify CD4+ and CD8+ lymphocytes populations from bronchoalveolar lavage fluid (BALF) at week 16 post-s.c. vaccination (week 10 post-i.n. vaccination).** The gating strategy shown corresponds to animal 368, vaccinated with the prime-boost HIMBpar-HIMBmuc vaccination strategy. T-cells were stimulated with PPDB and RPMI was used for unstimulated controls.

**
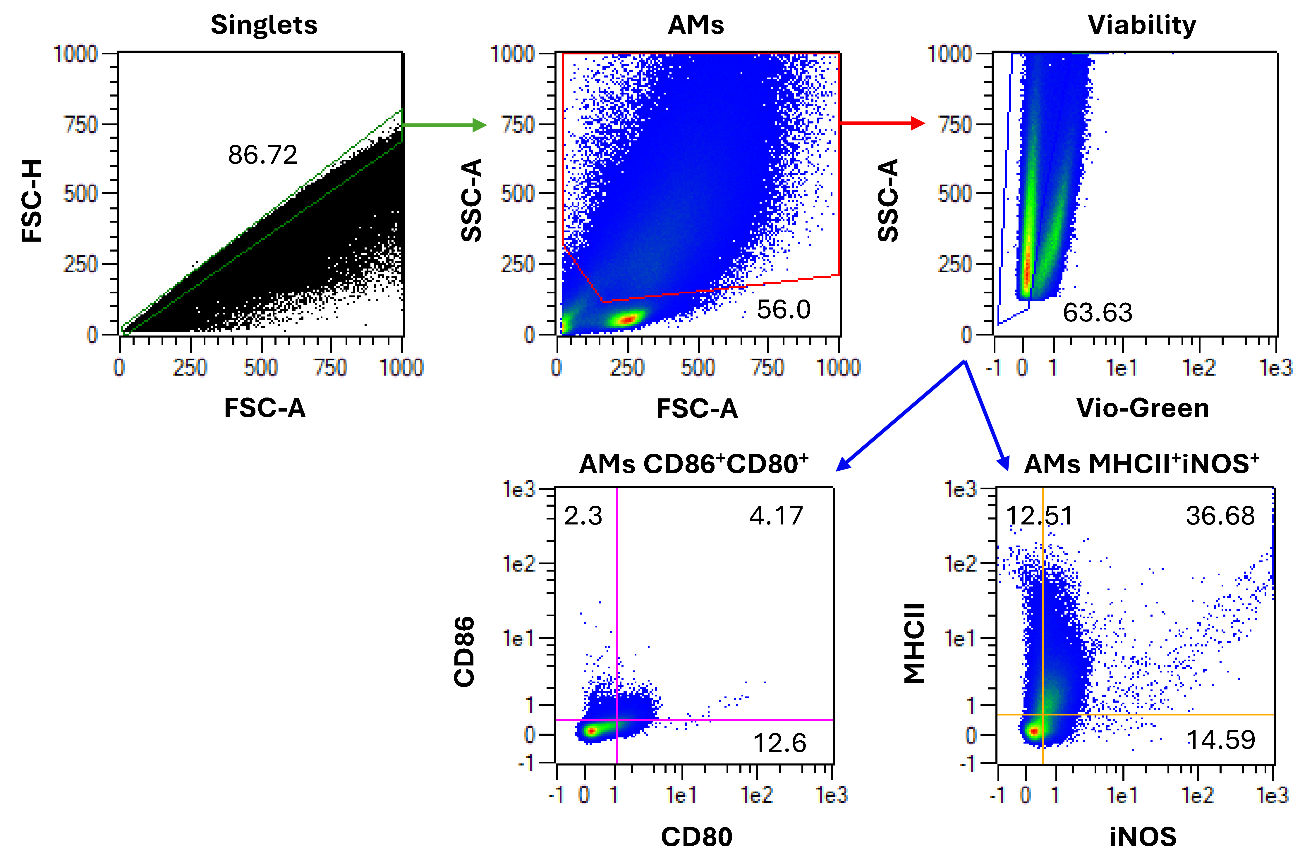
**

**Figure S3. Gating strategy used to identify alveolar macrophages (AMs) populations isolated form bronchoalveolar lavage fluid (BALF) at week 16 post-s.c. vaccination (week 10 post-i.n. vaccination).** The gating strategy shown corresponds to animal 353, vaccinated with the single-dose i.n. BCG strategy.

**
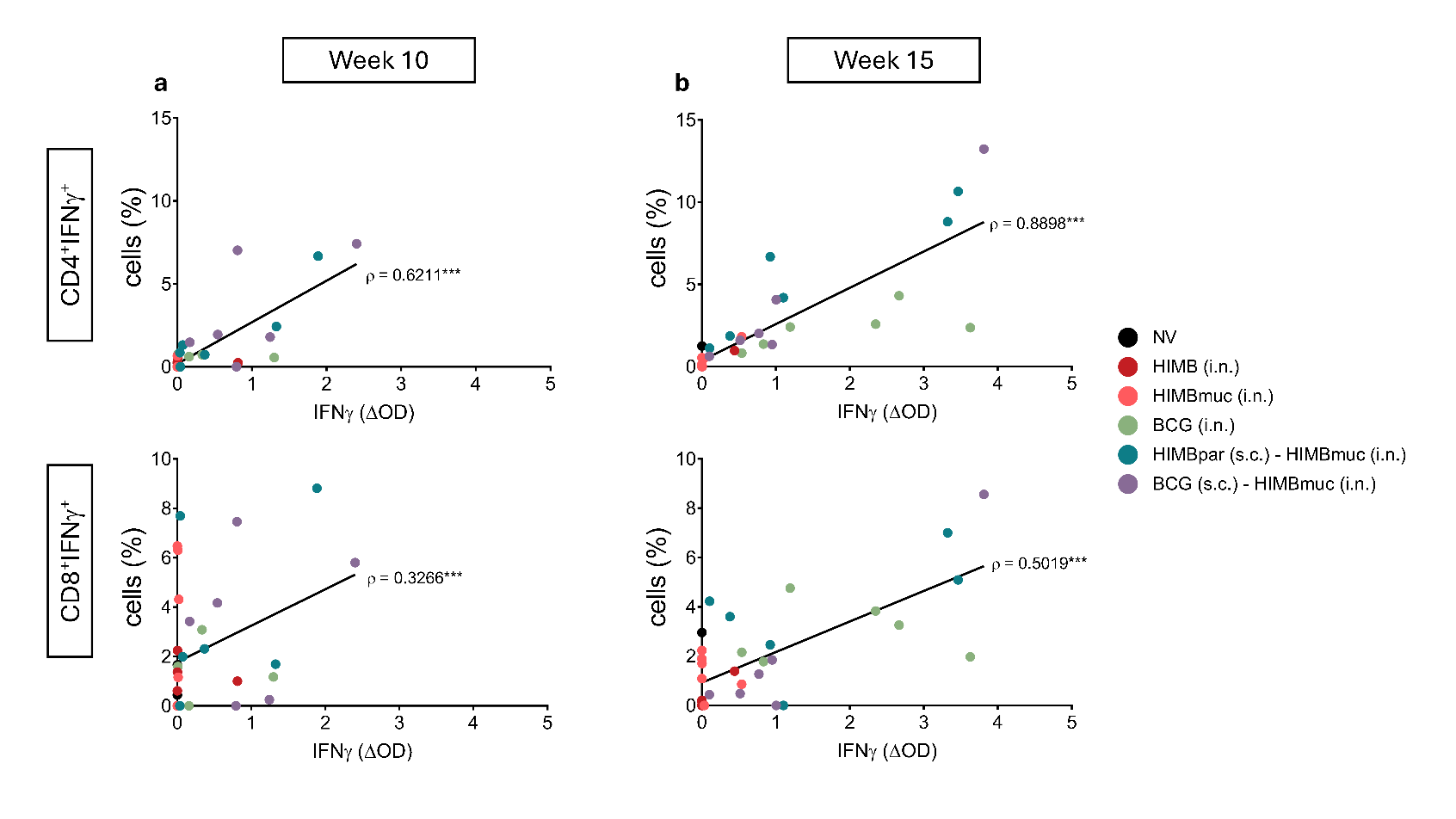
**

**Figure S4. Correlation between lymphocytes populations obtained from peripheral blood mononuclear cells (PBMCs) and IFNγ production by PBMCs measured by ELISA.** Correlation of CD4^+^IFNγ^+^ and CD8^+^IFNγ^+^ lymphocyte subsets frequencies with IFNγ released after PBMCs stimulation with PPD-B at week 10 (**a**) and week 15 (**b**) after s.c. vaccination (week 4 and week 9 after i.n. vaccination, respectively). PPD-B-specific cell frequencies (%) were calculated subtracting expression levels in PPDB-stimulated samples from those measured in non-stimulated samples. IFNγ (OD) levels were measured by ELISA with the ID Screen® Ruminant IFN-g kit (ID, Grabels, France), and OD levels were calculated as OD PPDB – OD PBS. ** *p* < 0.01, **** *p* < 0.0001 (two-tailed Spearman (ρ)). Each colour represents a vaccination group; each dot represents an individual. Prime vaccination was administered subcutaneously at week 0 (HIMBpar s.c. or BCG s.c.), followed by intranasal boost (HIMBmuc i.n.) and single-dose intranasal (BCG i.n., HIMBmuc i.n., HIMB i.n., and HIMBmuc i.n.) vaccination at week 6.


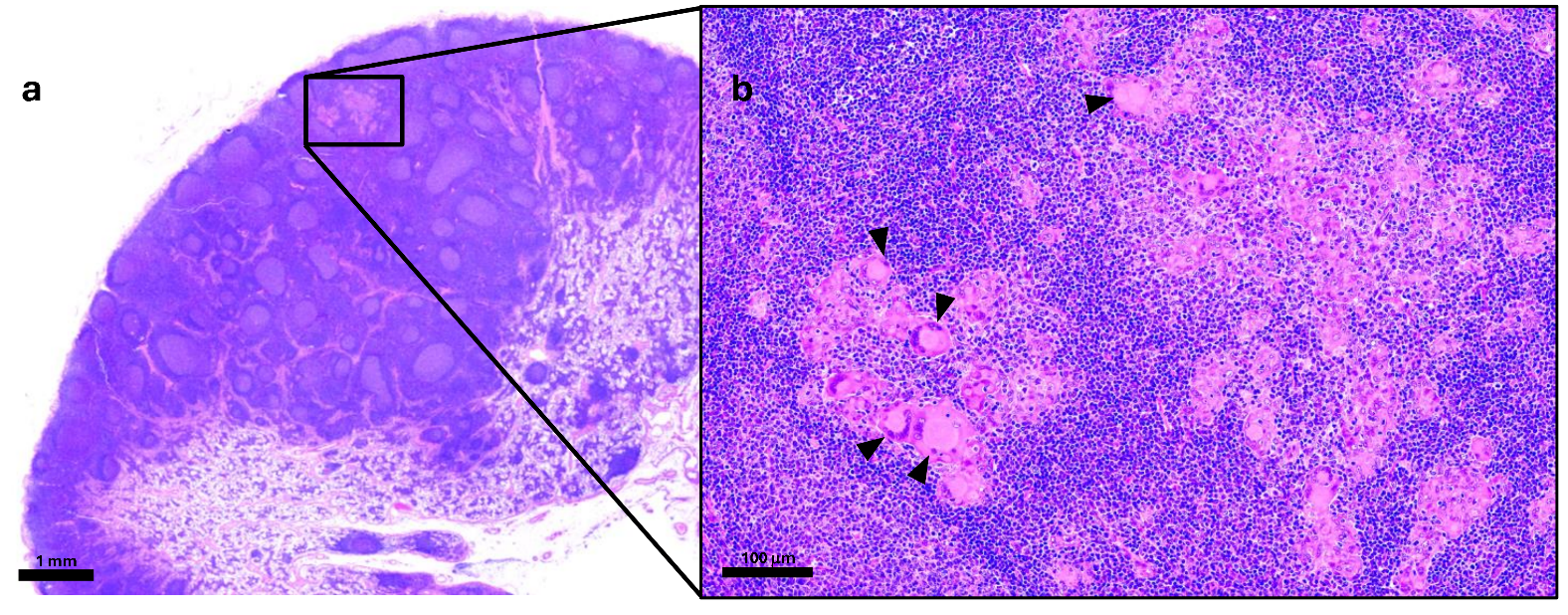
**Figure S5. Histopathological features of the left retropharyngeal lymph node from an intranasally BCG-vaccinated goat.** (**A**) Tissue section from the left retropharyngeal lymph node from goat 380 vaccinated with BCG (intranasal). Scale bar = 1 mm. (**B**) Focal granulomatous lesion with multinucleated giant cells (Langhans cells) (black arrowheads). Scale bar = 100 μm. Hematoxylin and eosin (H&E) staining.

Supplementary Table S1. Flow cytometry panel.

| Cytometry | Marker | Conjugate | Isotype | Clone | Source |
| --- | --- | --- | --- | --- | --- |
| Matrix | Live/Dead | Viobility 405/520 | - | Fixable Dye | Miltenyi Biotec |
| T-cell cytometry -from PBMCs | Goat, Sheep CD4 | FITC | Mouse, IgG2a | 44.38 | ThermoFisher Scientific |
|  | Sheep CD8 | Unconjugated  NovaFluor 755 | Mouse, IgG2A | 38.65 | Bio-Rad |
|  |  |  |  |  | ThermoFisher Scientific |
|  | Bovine CD62L | Unconjugated  Alexa Fluor 405 | Mouse IgG1 | IVA94 | ThermoFisher Scientific |
|  |  |  |  |  |  |
|  | Bovine, goat IFNγ | Unconjugated  Alexa Fluor 647 | Mouse IgG1 | CC302 | ThermoFisher Scientific |
|  |  |  |  |  |  |
|  |  |  |  |  |  |
| AMs cytometry from BALF | Bovine CD80 | FITC | Mouse, IgG1 | IL-A159 | ThermoFisher Scientific |
|  | Bovine CD86 | PE | Mouse, IgG1 | IL-A190 | ThermoFisher Scientific |
|  | Sheep CD163 | Unconjugated | Mouse, IgG1 | EDHu-1 | ThermoFisher Scientific |
|  | Mouse iNOS | Unconjugated  Alexa Fluor 647 | Mouse, IgG1 | 4E5 | ThermoFisher Scientific |
|  |  |  |  |  |  |
|  | Bovine MHCII | Unconjugated  NovaFluor 755 | Mouse, IgG2a | IL-A21 | ThermoFisher Scientific |
|  |  |  |  |  |  |
|  |  |  |  |  |  |
| T-cell cytometry from BALF | Goat, Sheep CD4 | PE | Mouse, IgG2a | 44.38 | ThermoFisher Scientific |
|  | Bovine, Goat, Sheep CD8 | FITC | Mouse, IgG2a | 38.65 | ThermoFisher Scientific |
|  | Bovine, goat IFNγ | Unconjugated  Alexa Fluor 647 | Mouse IgG1 | CC302 | ThermoFisher Scientific |
|  |  |  |  |  |  |

PBMCs, peripheral blood mononuclear cells; FITC, fluorescein isothiocyanate; AMs, alveolar macrophages; BALF, bronchoalveolar lavage fluid; PE, phycoerythrin; MHCII Major Histocompatibility Complex-II (MHCII); iNOS, inducible nitric oxide synthase. Miltenyi Biotec, Bergisch Gladbach, Germany; ThermoFisher Scientific, Waltham, Massachusetts, USA; Bio-rad, Hercules, California, USA.

Supplementary Table S2. Significant *p*-values corresponding to Fig. 2 (Whole-blood IFNγ responses throughout the study).

| Antigen | Week | p-value |  |  |  |
| --- | --- | --- | --- | --- | --- |
|  |  | * | ** | *** | **** |
| PPDB | W2 | HIMB vs. HIMBpar-HIMBmuc | BCG i.n. vs. BCG-HIMBmuc  HIMBmuc vs. HIMBpar-HIMBmuc | HIMB vs. BCG-HIMBmuc | HIMBmuc vs. BCG-HIMBmuc |
|  | W4 |  | BCG i.n., HIMB, HIMBmuc vs. BCG-HIMBmuc  BCG i.n., HIMB, HIMBmuc vs. HIMBpar-HIMBmuc |  |  |
|  | W6 |  | BCG i.n., HIMB, HIMBmuc vs. BCG-HIMBmuc  BCG i.n., HIMB vs. HIMBpar-HIMBmuc | HIMBmuc vs. HIMBpar-HIMBmuc |  |
|  | W8 | BCG i.n. vs. HIMBpar-HIMBmuc | BCG i.n., HIMB, HIMBmuc vs. BCG-HIMBmuc  HIMB vs. HIMBpar-HIMBmuc |  |  |
|  | W10 | HIMBmuc vs. BCG-HIMBmuc | HIMB i.n. vs. BCG-HIMBmuc  HIMB i.n., HIMBmuc vs. HIMBpar-HIMBmuc |  |  |
|  | W12 | HIMBmuc vs. BCG i.n.  HIMBmuc vs. BCG-HIMBmuc | HIMB vs. BCG i.n.  HIMB vs. BCG-HIMBmuc | HIMB, HIMBmuc vs. HIMBpar-HIMBmuc |  |
|  | W14 | HIMB, HIMBmuc vs. BCG i.n. | HIMB, HIMBmuc vs. BCG-HIMBmuc | HIMB, HIMBmuc vs. HIMBpar-HIMBmuc |  |
|  | W16 | HIMBmuc vs. BCG-HIMBmuc | HIMB, HIMBmuc vs. BCG i.n.  HIMB vs. BCG-HIMBmuc | HIMB, HIMBmuc vs. HIMBpar-HIMBmuc |  |
| ESAT-6  CFP-10  Rv3615c | W2 | HIMB, HIMBmuc vs. HIMBpar-HIMBmuc | BCG i.n. vs. HIMBpar-HIMBmuc |  |  |
|  | W4 |  | BCG i.n., HIMB, HIMBmuc, BCG-HIMBmuc vs. HIMBpar-HIMBmuc |  |  |
|  | W6 |  |  |  |  |
|  | W8 | HIMB vs. HIMBpar-HIMBmuc |  |  |  |
|  | W10 | HIMBmuc, BCG-HIMBmuc vs. HIMBpar-HIMBmuc  BCG i.n. vs. HIMB |  | HIMB vs. HIMBpar-HIMBmuc |  |
|  | W12 |  | HIMB, BCG-HIMBmuc vs. HIMBpar-HIMBmuc | HIMBmuc vs. HIMBpar-HIMBmuc |  |
|  | W14 | HIMB, HIMBmuc vs HIMBpar-HIMBmuc  HIMB, HIMBmuc vs. BCG-HIMBmuc |  | BCG i.n. vs. HIMBpar-HIMBmuc | BCG-HIMBmuc vs. HIMBpar-HIMBmuc |
|  | W16 | HIMBmuc vs. HIMBpar-HIMBmuc | BCG i.n., BCG-HIMBmuc vs. HIMBpar-HIMBmuc | HIMB vs. HIMBpar-HIMBmuc |  |

PPDB, *M. bovis* purified protein derivative; **p* < 0.05, ** *p* < 0.01, ****p* < 0.001, **** *p* < 0.0001.

Supplementary Table S3. Significant *p*-values corresponding to Fig. 4 (Serum MTBC-specific IgG responses throughout the study).

| Week | p-value |  |  |
| --- | --- | --- | --- |
|  | * | ** | *** |
| W2 | BCG i.n., HIMB vs. BCG-HIMBmuc |  |  |
| W4 | HIMB, HIMBmuc vs. BCG-HIMBmuc | BCG i.n. vs. BCG-HIMBmuc  HIMB vs. HIMBpar-HIMBmuc | BCG i.n., HIMBmuc vs. HIMBpar-HIMBmuc |
| W6 | HIMBmuc vs. BCG-HIMBmuc | BCG vs. BCG-HIMBmuc  HIMB vs. HIMBpar-HIMBmuc | BCG i.n., HIMBmuc vs. HIMBpar-HIMBmuc |
| W8 | BCG i.n., HIMBmuc vs. BCG-HIMBmuc | HIMB vs. HIMBpar-HIMBmuc | BCG i.n., HIMBmuc vs. HIMBpar-HIMBmuc |
| W10 | BCG i.n., HIMBmuc vs. BCG-HIMBmuc | HIMB vs. HIMBpar-HIMBmuc | BCG i.n., HIMBmuc vs. HIMBpar-HIMBmuc |
| W12 | BCG-HIMBmuc vs. HIMBpar-HIMBmuc | HIMB, HIMBmuc vs. HIMBpar-HIMBmuc | BCG i.n. vs. HIMBpar-HIMBmuc |
| W14 | HIMB vs. HIMBpar-HIMBmuc |  | BCG i.n., HIMBmuc vs. HIMBpar-HIMBmuc |
| W16 | BCG i.n. vs. HIMBpar-HIMBmuc | HIMB, HIMBmuc vs. BCG-HIMBmuc | HIMB, HIMBmuc vs. HIMBpar-HIMBmuc |

**p* < 0.05, ** *p* < 0.01, ****p* < 0.001
